# Supplementary material for: Evolutionary and functional genomics of DNA methylation in maize domestication and improvement
Source: Nat Commun. 2020 Nov 2;11:5539. doi: 10.1038/s41467-020-19333-4 (PMC7606521; doi:10.1038/s41467-020-19333-4)
Supplement: Supplementary file 1 — Supplementary Information [file 41467_2020_19333_MOESM1_ESM.pdf]

Evolutionary and functional genomics of DNA methylation in maize domestication and improvement

Xu *et al.*

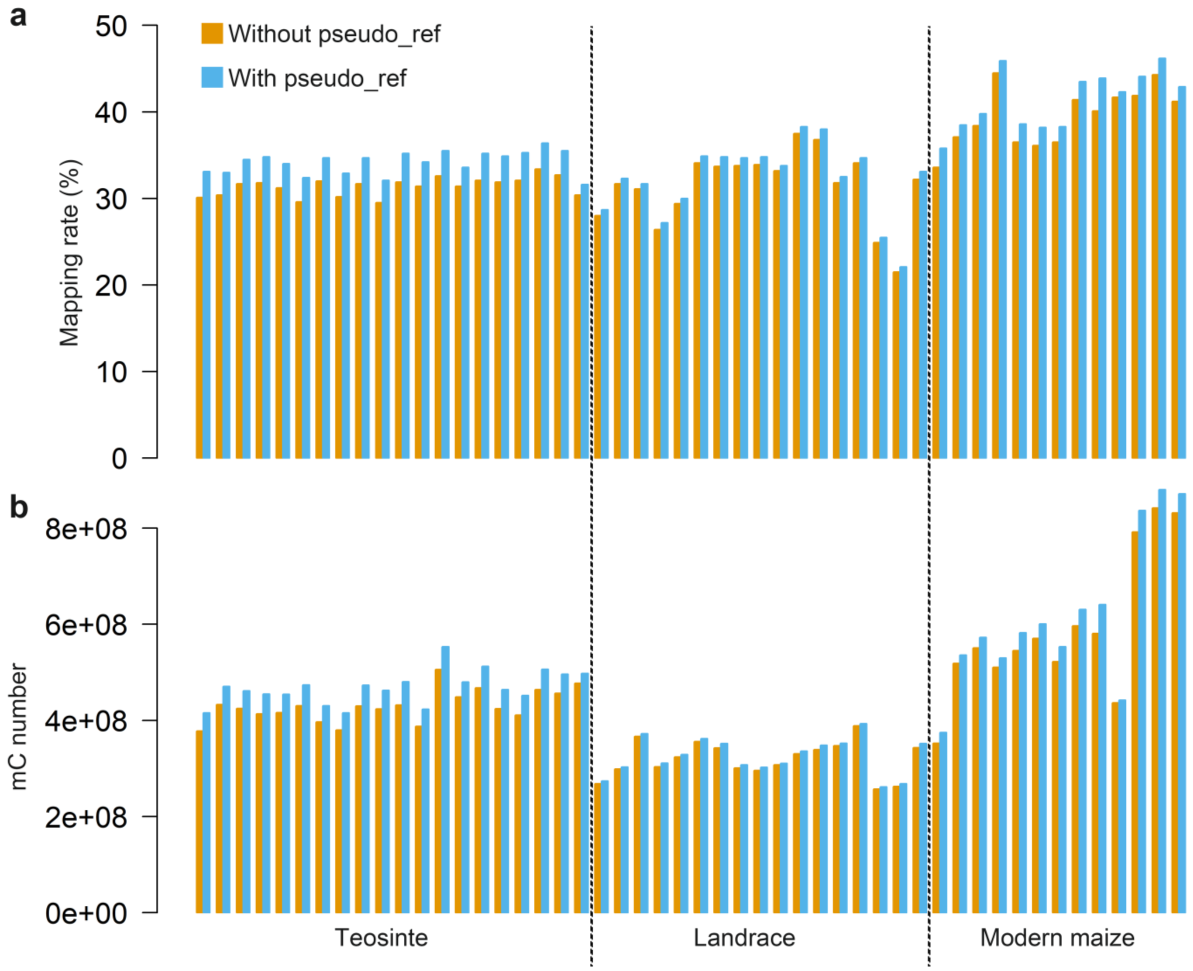

Supplementary Fig. 1. Comparison of mapping rates and number of methylated cytosine sites. The mapping rates (**a**) and the number of methylated cytosine (mC) sites (**b**) with and without using pseudo reference genome in different populations. B73 reference genome (AGPv4) was used in the analyses. Source data are provides as a Source Data file.

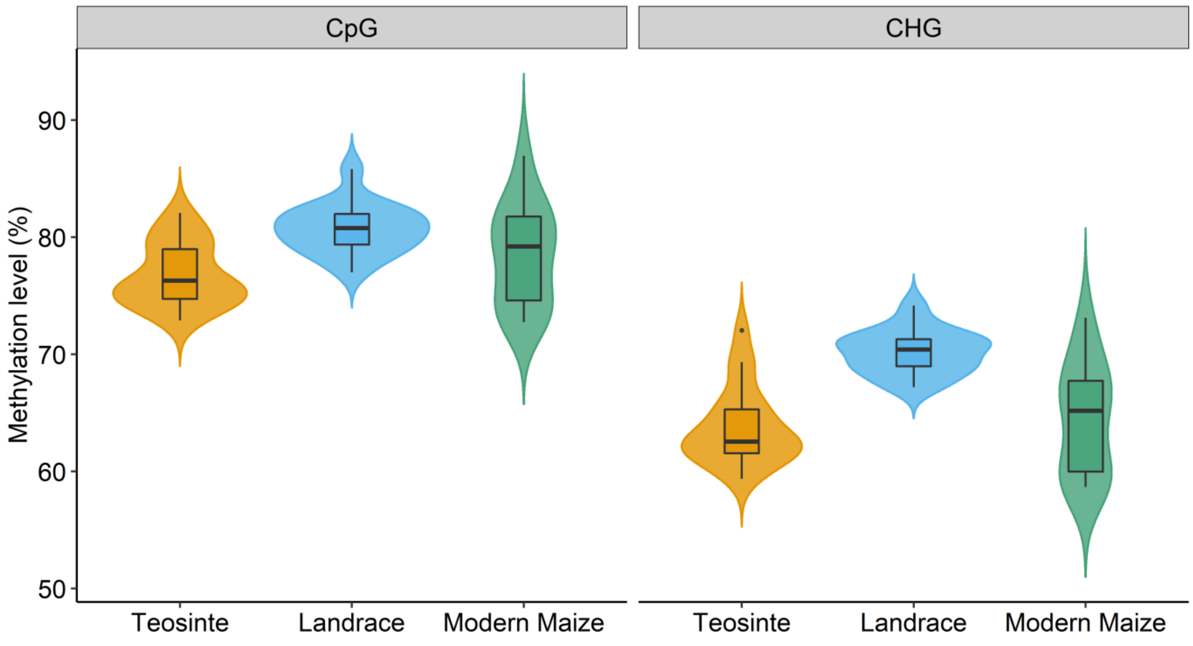

Supplementary Fig. 2. Distributions of levels of DNA methylation in teosinte, landrace, and modern maize populations. Left panel denotes results for CG sites and right panel denotes results for CHG sites. Source data are provides as a Source Data file.

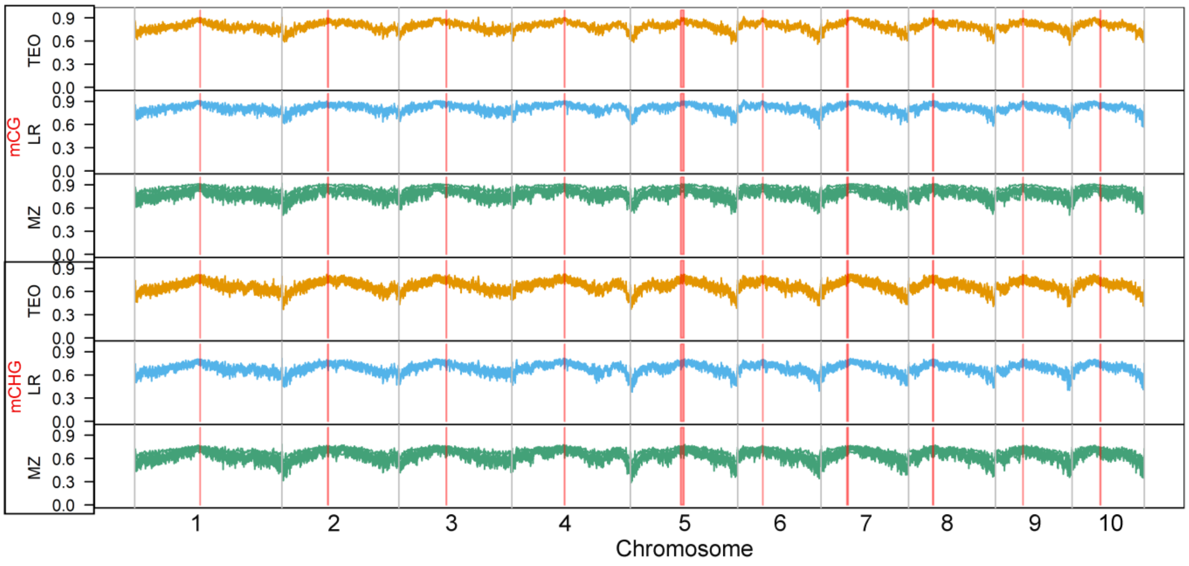

Supplementary Fig. 3. Genome-wide distributions of DNA methylation across 10 maize chromosomes. TEO, LR, and MZ represent teosinte, landrace and modern maize populations, respectively. Red vertical lines indicate the pericentromeric regions. Source data are provides as a Source Data file.

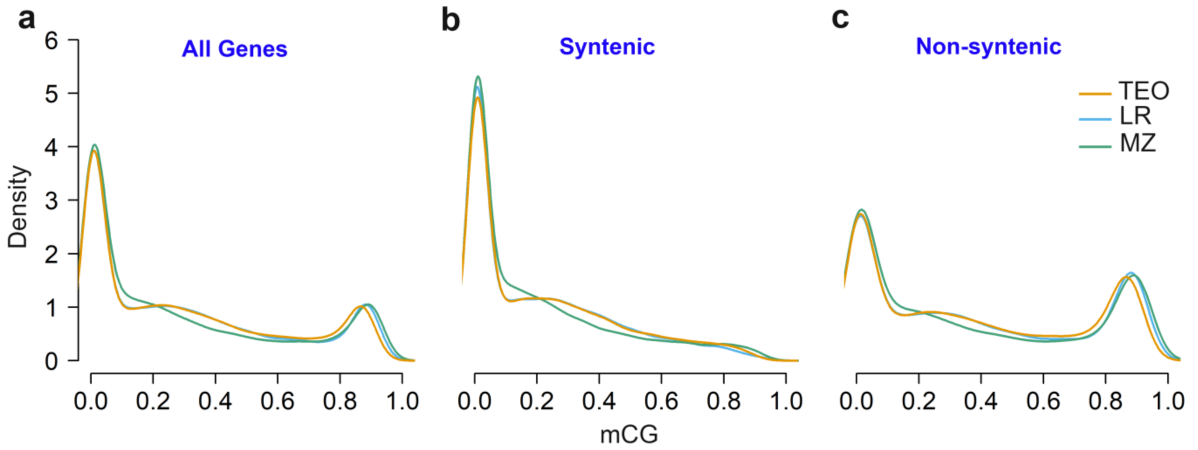

Supplementary Fig. 4. Density plots of CG methylation in gene body. Panels represent for all the annotated maize genes (**a**), syntenic genes (**b**), and nonsyntenic genes (**c**). TEO, LR, and MZ represent teosinte, landrace, and modern maize populations, respectively. The syntenic and nonsyntenic orthologs were determined by comparing maize with sorghum. Source data are provides as a Source Data file.

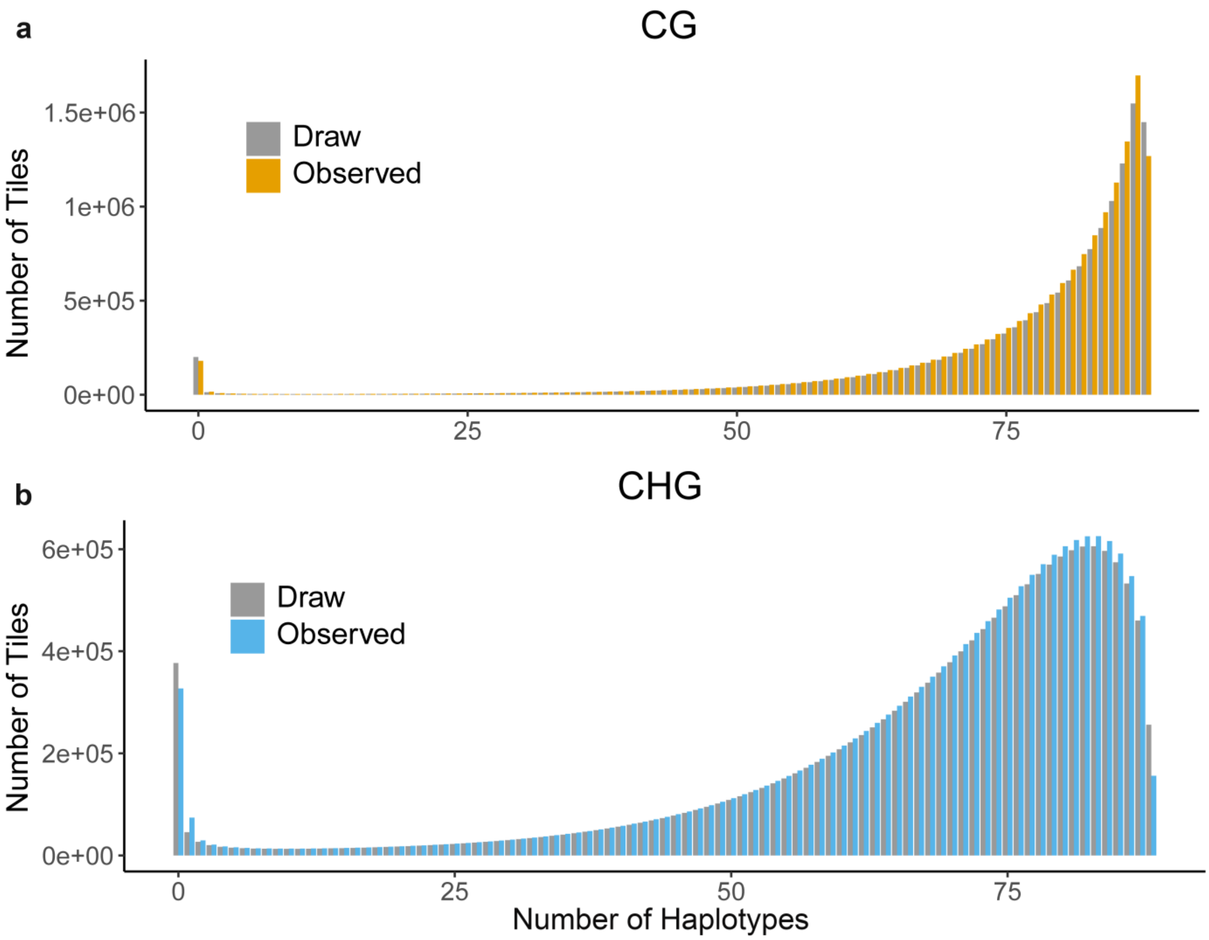

Supplementary Fig. 5. Observed and posterior methylome site frequency spectra. The posterior methylome site frequency spectra (mSFS) was calculated in CG (**a**) and CHG (**b**) context using parameters drawn from the 1,000,000th iteration (*Ne* = 50,000).

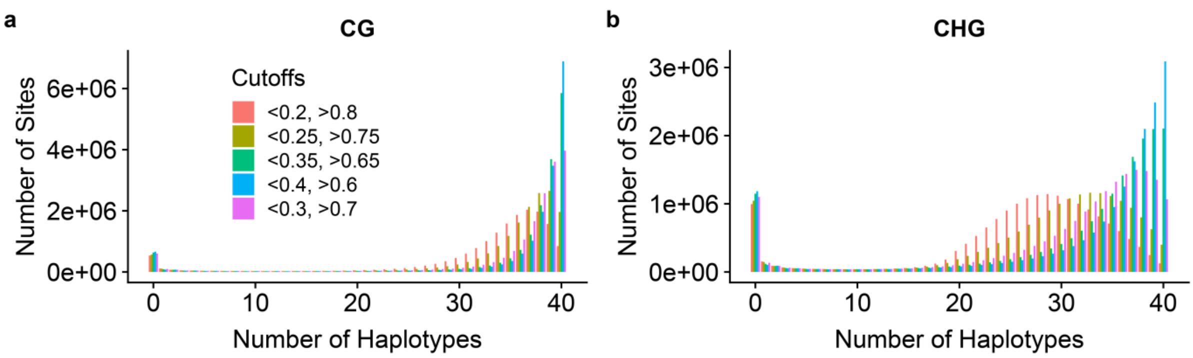

Supplementary Fig. 6. Sensitivity tests using different cutoffs. Distributions of methylome site frequency spectra (mSFS) using different thresholds to determine the methylated, unmethylated, and heterozygotic100-bp tiles under CG (**a**) and CHG (**b**) contexts.

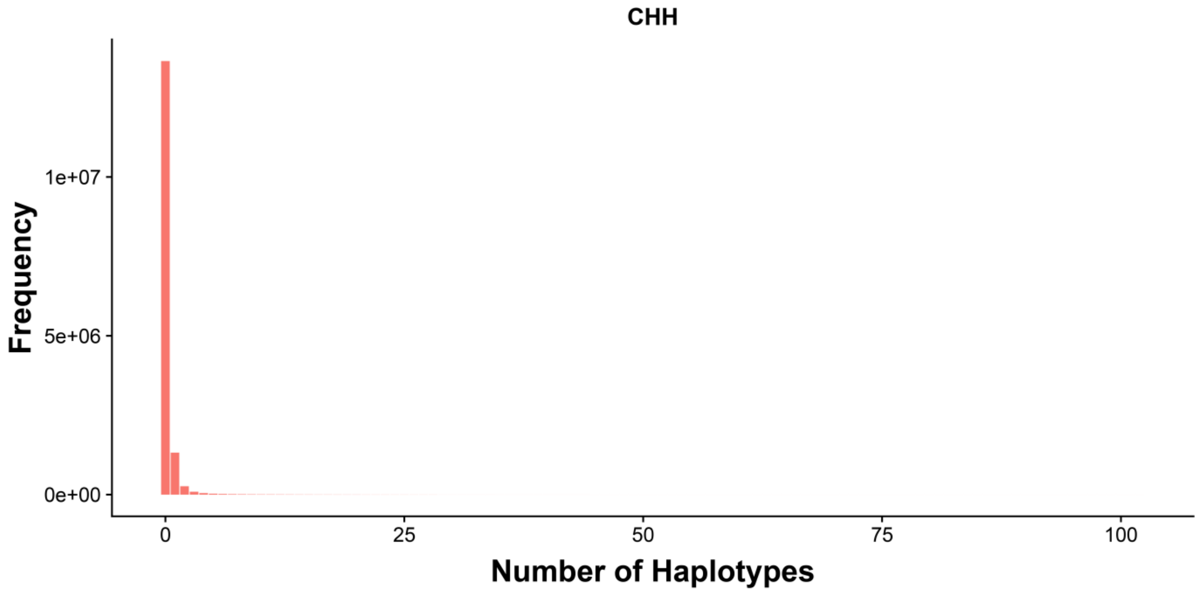

Supplementary Fig. 7. Methylome site frequency spectrum under the CHH context. The distribution is highly skewed towards the unmethylated status for CHH sites.

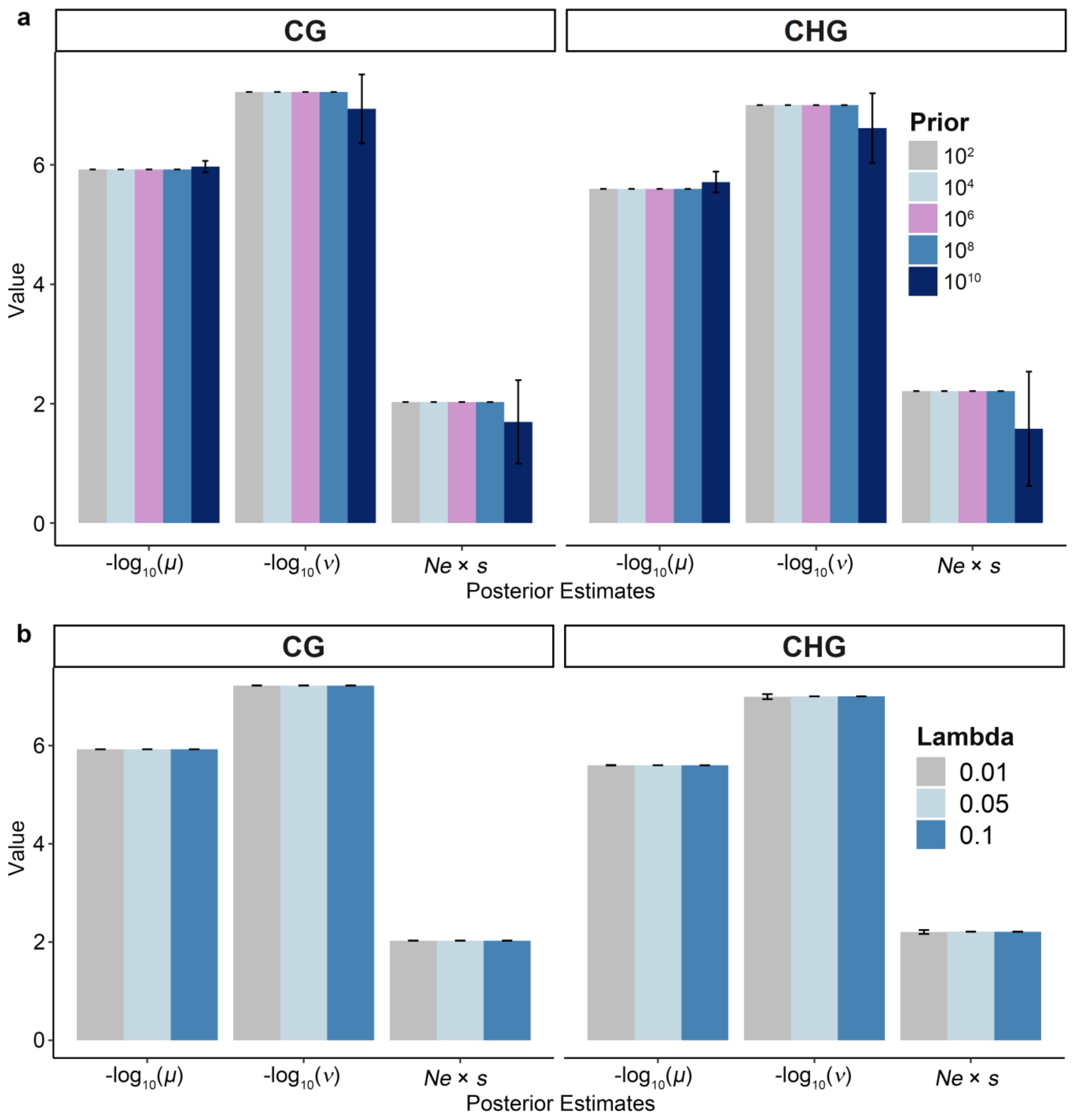

Supplementary Fig. 8. The effect of prior values on posterior parameter estimations. (**a**) Prior values of 10^2, 10^4, 10^5, 10^8, and10^{10} were used for the exponential proposal distributions. (**b**) Lambda values of the scaled proposal distribution of 0.01, 0.05, and 0.1 were used. Error bars indicate standard deviations (*N* = 1,600 for each bar). Source data are provides as a Source Data file.

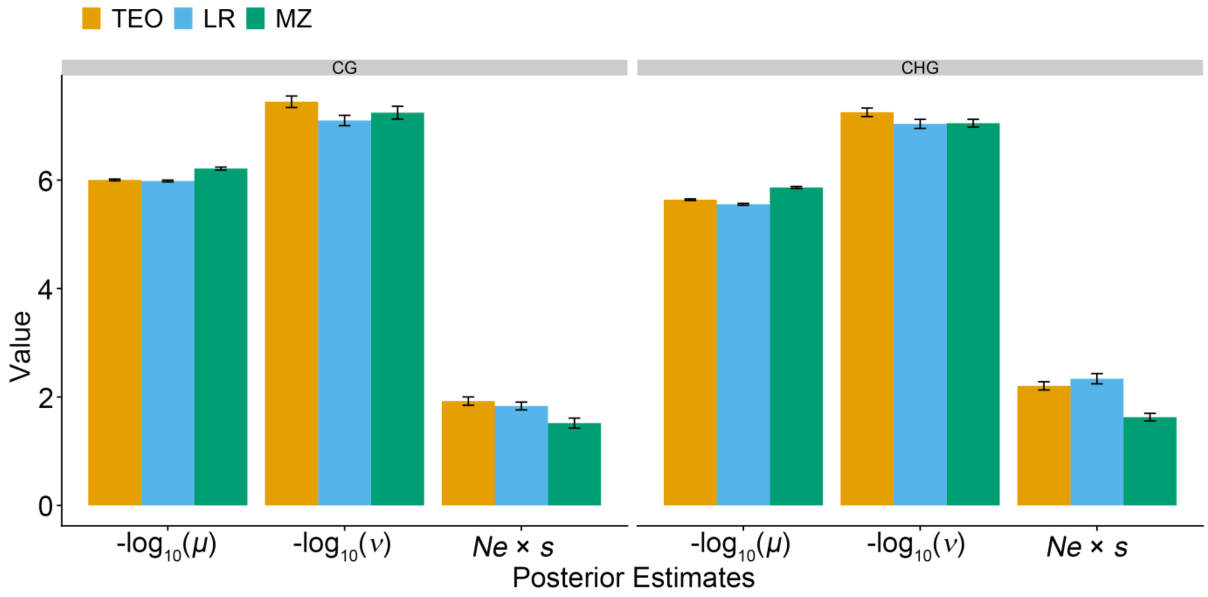

Supplementary Fig. 9. Population genetic parameter inference using each individual population. Posterior estimators of mean values and standard deviations for μ, ν, and $Ne \times s$ for CG and CHG sites. Values were estimated using MCMC approach with 25% burnin. Error bars indicate standard deviations (N = 1,600 for each bar). Source data are provides as a Source Data file.

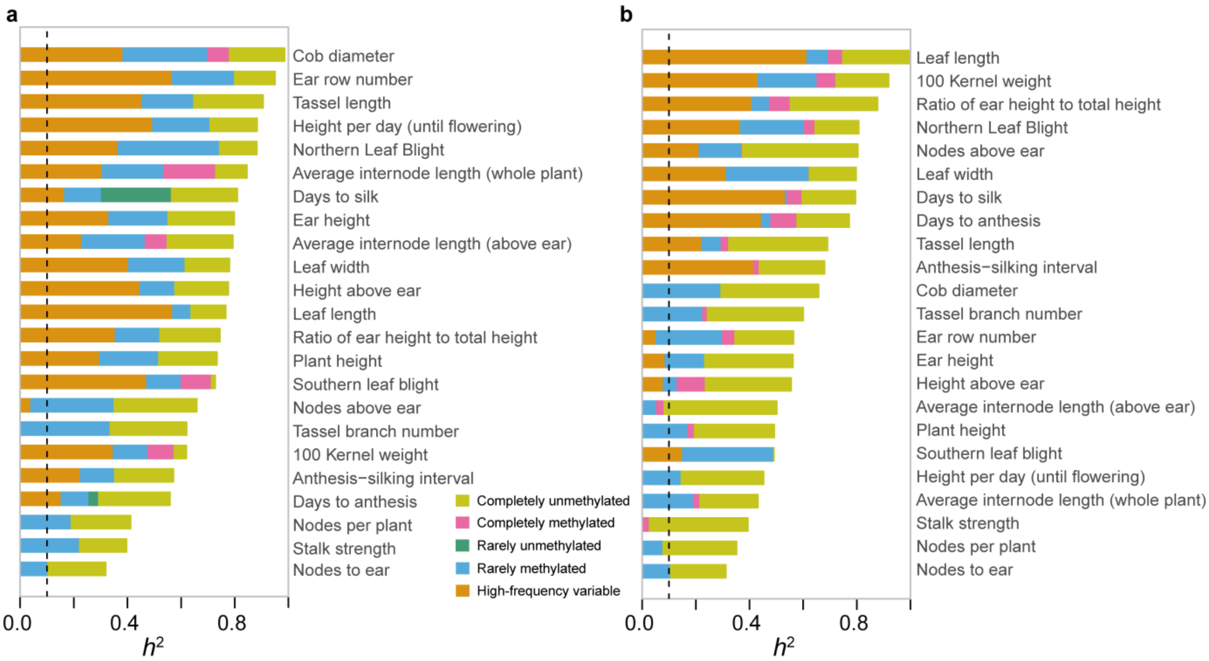

Supplementary Fig. 10. Proportion of genetic variances explained by SNP subsets residing in different genomic regions with different DNA methylation status. The proportion of genetic variance explained (h^2) by different SNP subsets under CG (**a**) and CHG (**b**) contexts. Source data are provides as a Source Data file.

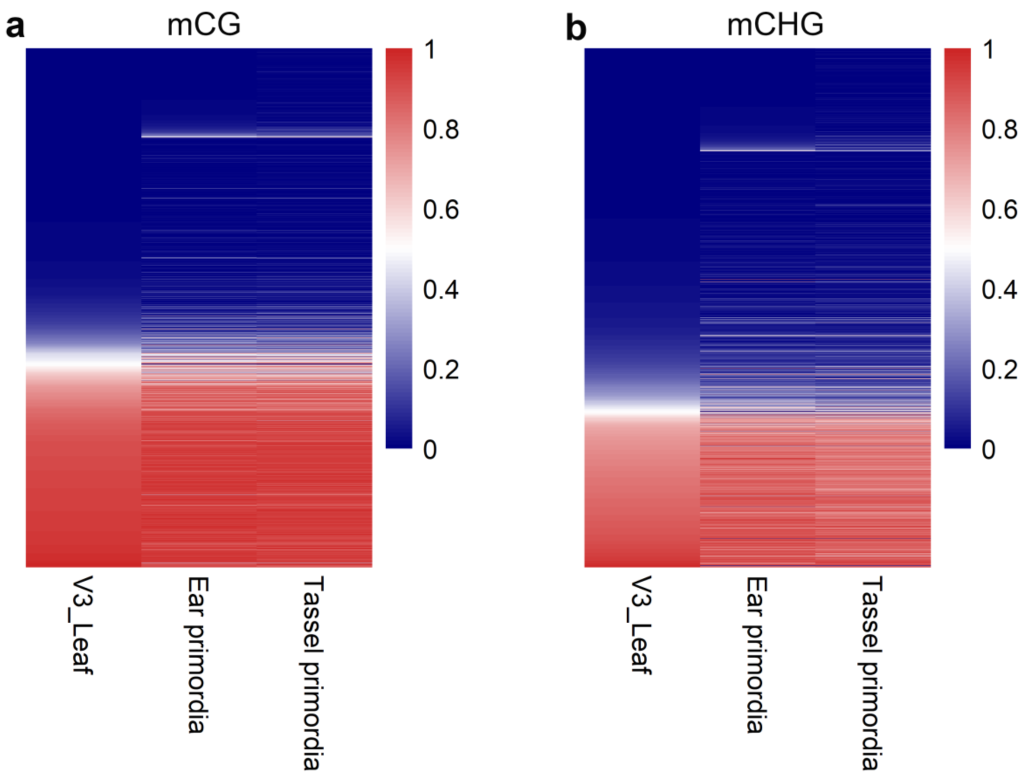

Supplementary Fig. 11. The methylation patterns of the differentially methylated regions across different tissues. DNA methylation levels in CG (**a**) and CHG (**b**) of each DMR across three tissues. The colors in the heat map indicate the high (red) or low (blue) DNA methylation levels. Source data are provides as a Source Data file.

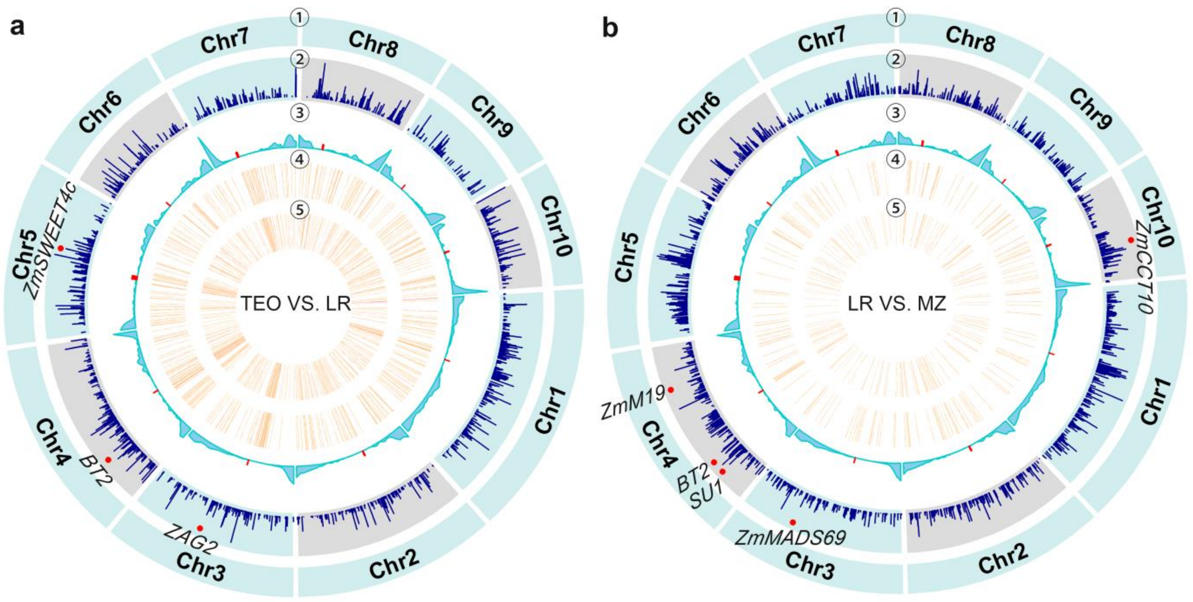

Supplementary Fig. 12. Landscape of selection signals and DNA methylation variation across maize genome. Genome-wide distributions of selective sweeps, DMRs across ten maize chromosomes detected by teosinte vs. landrace (**a**) and landrace vs. modern maize (**b**). From outer to inner circles are ① chromosome names, ② selective sweeps, ③ recombination rate, and the density of DMRs (number per 1-Mb) in ④ CG and ⑤ CHG contexts. Red dots at the second track indicate the physical positions of the known genes located within the selective sweeps. Red ticks at the third track indicate the centromeric regions.

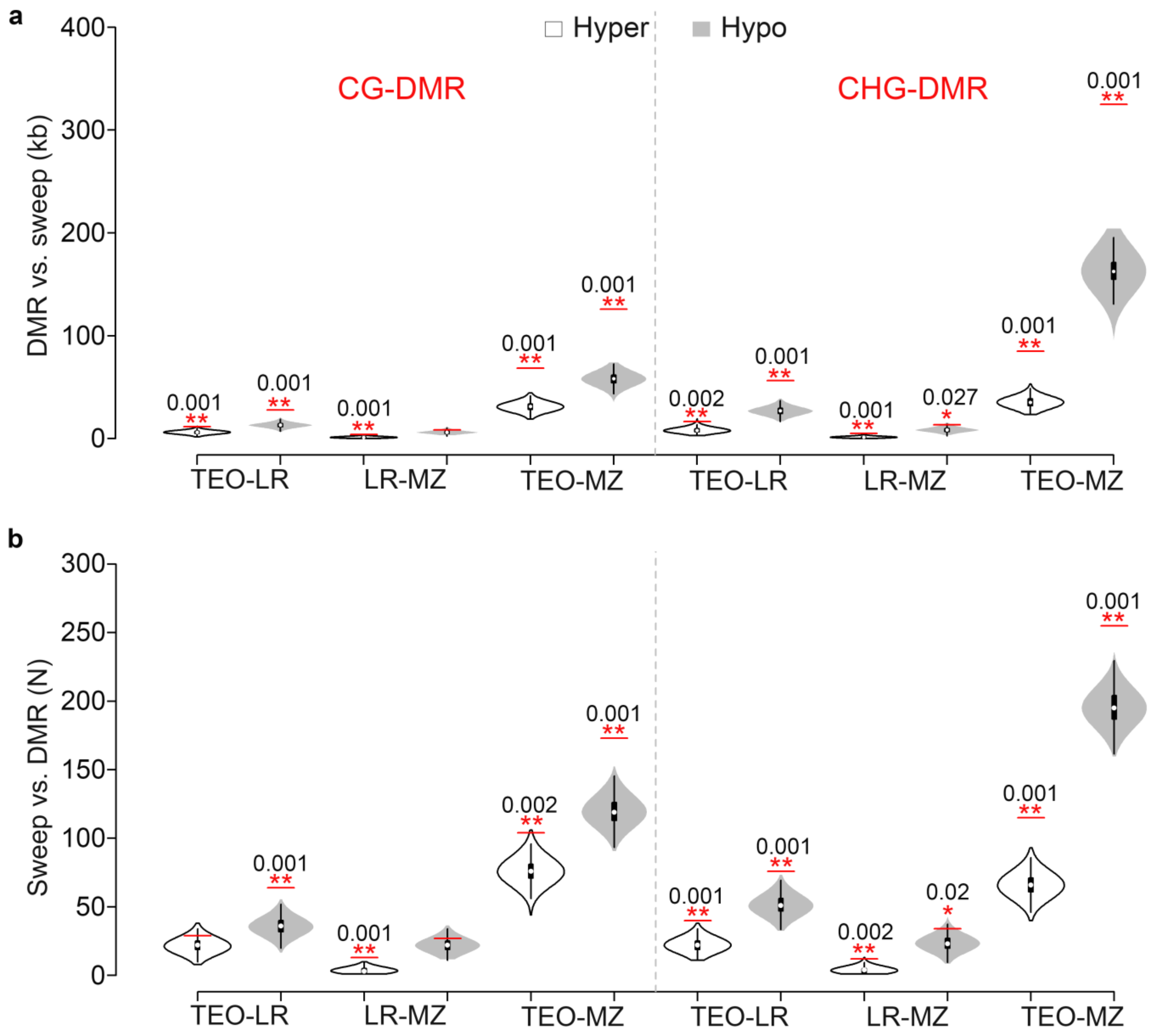

Supplementary Fig. 13. Comparison between differentially methylated regions and selective sweeps. (**a**) The overlapped base-pairs between DMRs and selective sweeps. (**b**) The number of sweeps that overlapped with DMRs. Red horizontal bars indicate the observed values and violin plots showed the 1,000 one-sided permutation results using randomly selected mappable regions from the genome. Red asterisks indicate the statistical significance with one asterisk denoting *P*-value < 0.05 and two asterisks denoting *P*-value < 0.01. The numbers above the asterisks indicate the exact *P*-value. Hyper- and hypomethylation were defined based on maize.

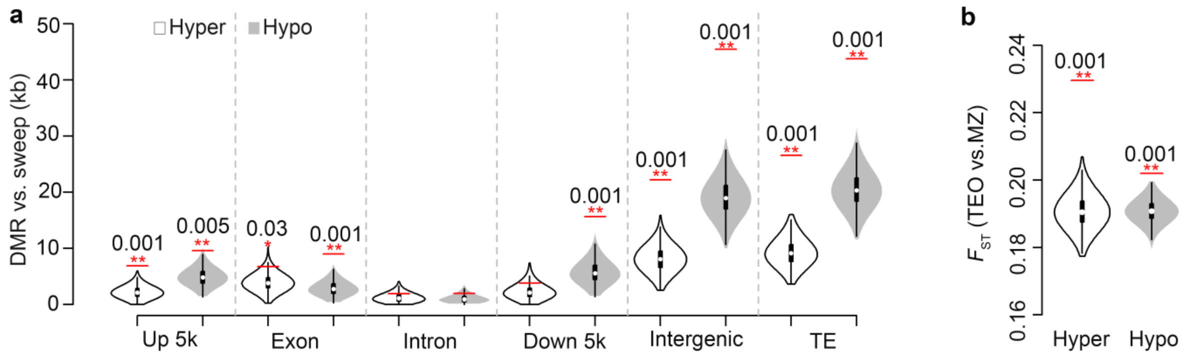

Supplementary Fig. 14. Selection on differentially methylated regions. (**a**) Overlaps between teosinte-maize DMRs and selective sweeps breaking down into different genomic features. (**b**) Mean F_{ST} values of teosinte-maize DMRs that were hyper- and hypomethylated in maize. Red horizontal bars indicate the observed values and violin plots show the 1,000 one-sided permutation results using randomly selected regions sharing the similar genomic features as the DMRs. Red asterisks indicate the statistical significance with one asterisk denoting P-value < 0.05 and two asterisks denoting P-value < 0.01. The numbers above the asterisks indicate the exact P-value.

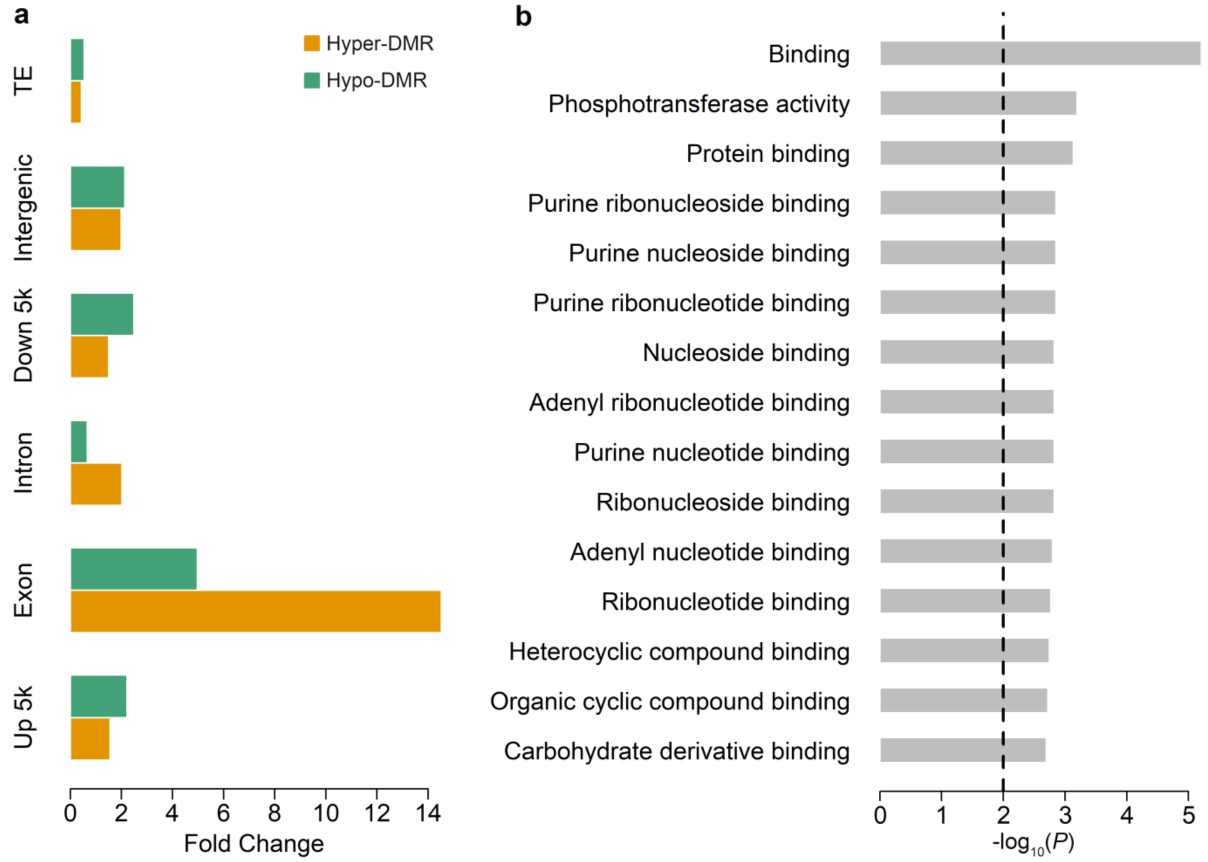

Supplementary Fig. 15. Teosinte-maize CG differentially methylated regions and their associated functional features. (**a**) Fold changes of mappable DMR length relative to the mean values from 1,000 permutations. (**b**) The results of gene ontology (GO) term enrichment test using genes exhibiting an exonic DMR. Vertical dashed line indicates the significance cutoff (Fisher's exact test, *P*-value = 0.01). Source data are provides as a Source Data file.

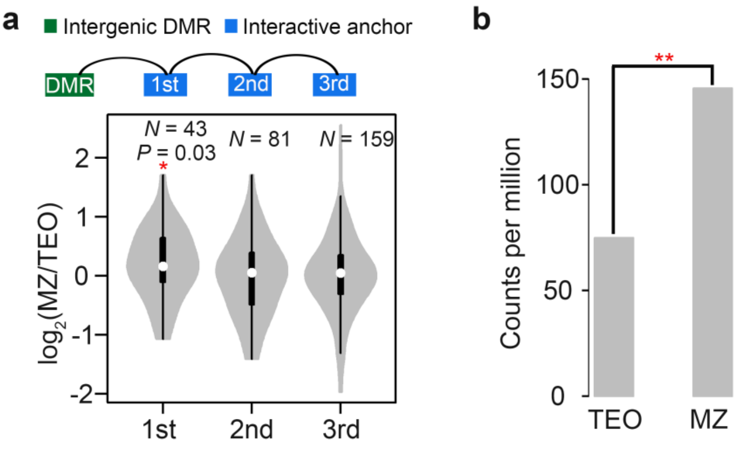

Supplementary Fig. 16. Intergenic CG differentially methylated regions altered downstream gene expression. (**a**) Contrast of the gene expression levels in maize relative to teosinte. In the upper panel, the schematic diagram shows the genes that involved in the 1st, 2nd, and 3rd level interactions with maize hypomethylated DMRs located in intergenic regions. Red asterisk indicates the statistically significant difference using two-sided paired *t*-test (*P*-value < 0.05). (**b**) Gene expression level of *Zm00001d018036* in teosinte and modern maize (Binomial test, *P*-value = 4.6×10^{-141}), with two red asterisks denoting *P*-value < 0.01.

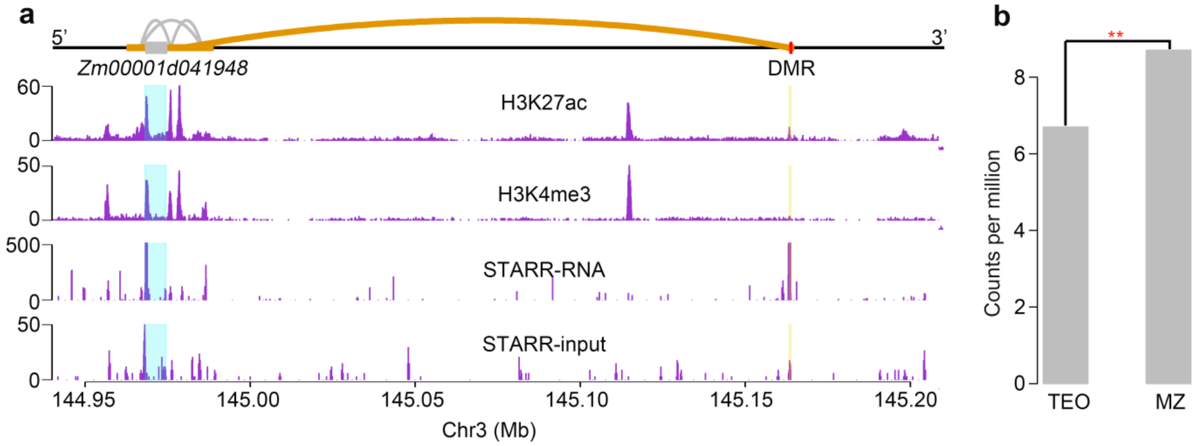

Supplementary Fig. 17. Interactive loops between a differentially methylated region and a gene model *Zm0001d041948*. (**a**) Chromatin interactions (the upper panel) and ChIP-Seq profiles (the lower panels) at gene *Zm00001d041948*. Gray and red boxes indicate the physical position of the gene model and the DMR. Gray and blue lines denote the interactive loops. (**b**) Gene expression level of *Zm00001d041948* in teosinte and modern maize (Binomial test, *P*-value = 4.3×10^{-3}), with two red asterisks denoting *P*-value < 0.01.

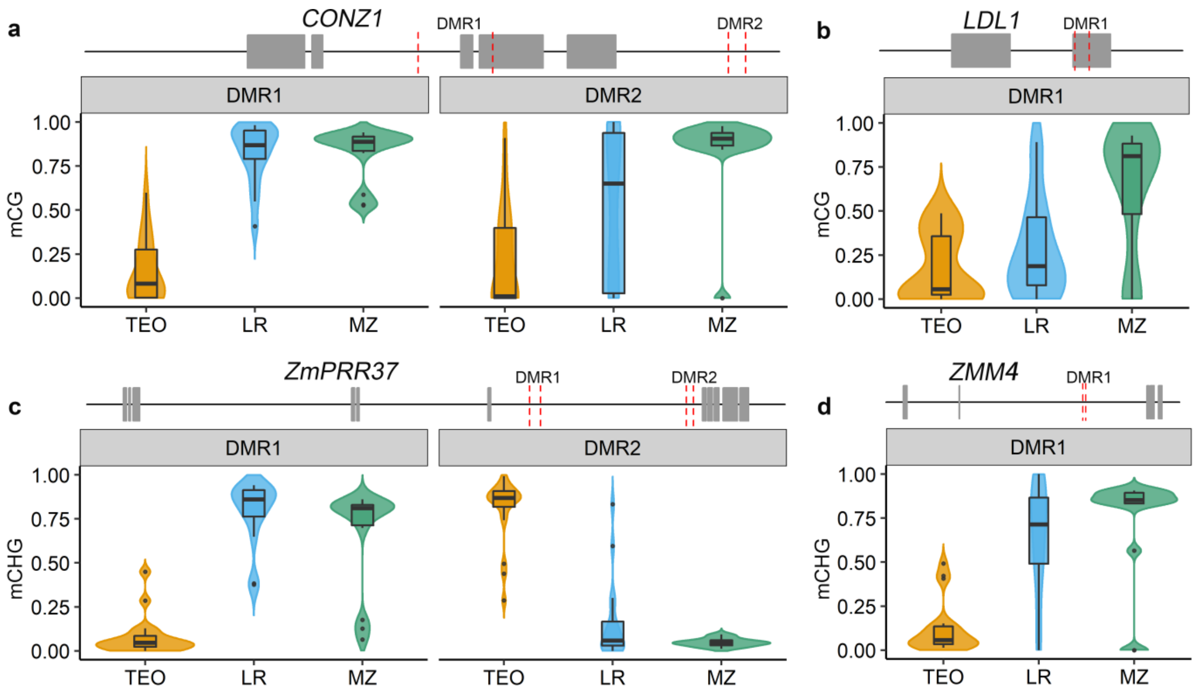

Supplementary Fig. 18. Teosinte-maize differentially methylated regions located at flowering time genes. Distribution of methylation levels within differentially methylated regions (DMRs) locating at *CONZ1* (**a**), *LDL1* (**b**), *ZmPRR37* (**c**) and *ZMM4* (**d**) in teosinte (TEO), landrace (LR), and modern maize (MZ). Two nearby vertical dashed red lines on the gene model indicate a teosinte-maize DMR. Source data underlying Supplementary Figure 18b are provides as a Source Data file.

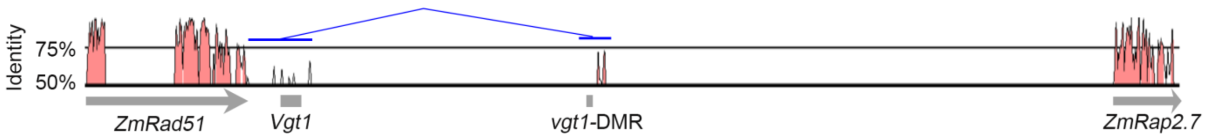

Supplementary Fig. 19. Conserved non-coding sequences between the maize and sorghum orthologous around *vgt1*-DMR. Sequence identity of the maize sequence spanning *vgt1* and the two proximal genes with corresponding sorghum sequences. Red peaks denote the conserved non-coding sequences (CNSs) identified using a window size of 100 bp. The thin blue lines indicate physical interaction between two anchor sequences (horizontal thick blue lines)**.**

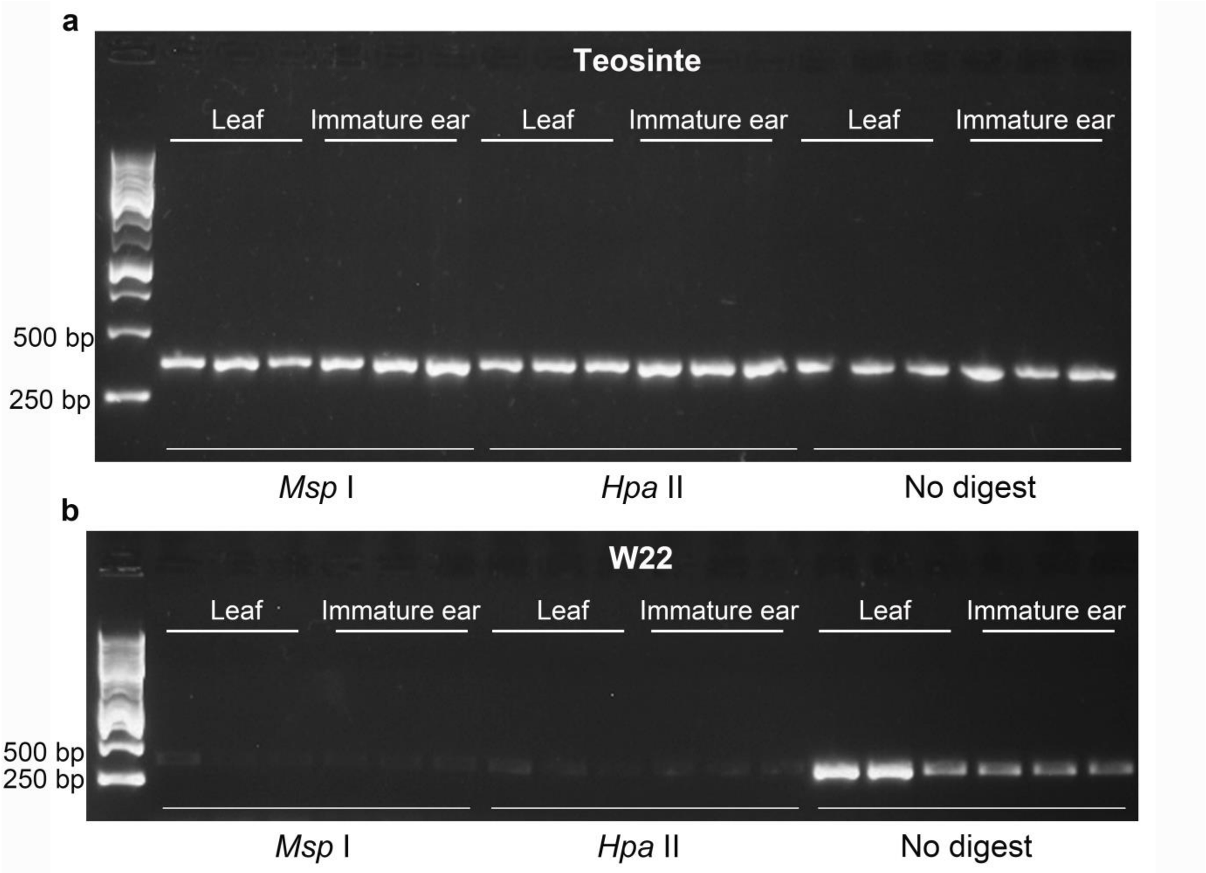

Supplementary Fig. 20. Experimental validation of the *tb1*-DMR using Chop-PCR. Chop–PCR analysis of *tb1*-DMR in different tissues of W22 (**a**) and teosinte 8759 (**b**). Failure to detect a PCR product suggests the loss of DNA methylation. CG methylation was detected using *Hpa*II and CHG methylation was detected using *Msp*I. Three independent biological replications are shown, each with three technical replications. No digested DNA was used as a control.

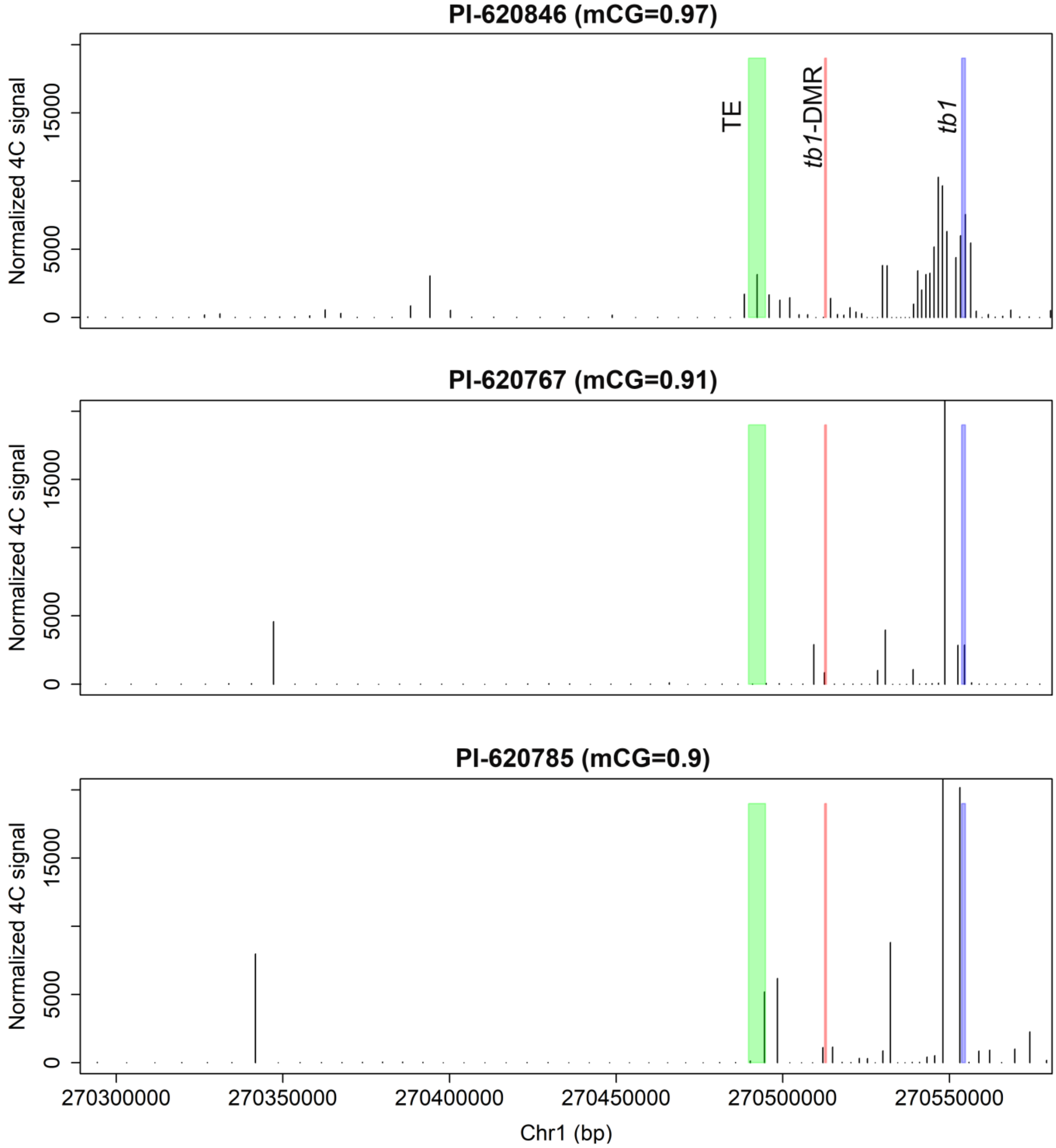

Supplementary Fig. 21. The 4C-seq results of regions interacted with *tb1* gene in *tb1*-DMR hypermethylated landrace samples. The titles show the accession names and CG methylation levels of the *tb1*-DMR. The green bar indicates the physical location of the *Hopscotch* TE; the red bar indicates the *tb1*-DMR; and the blue bar indicates the *tb1* gene.

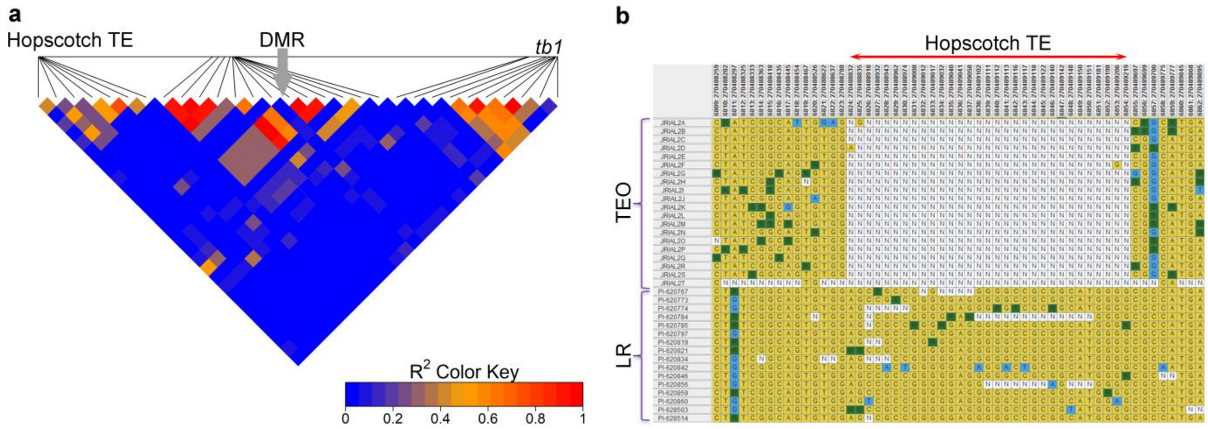

Supplementary Fig. 22. The linkage disequilibrium analysis among the *Hopscotch* transposon, *tb1*-DMR, and *tb1* gene. (**a**) The linkage disequilibrium (LD) heatmap using 17 landraces segregating at the *tb*1-DMR locus. The arrow indicates the position of the *tb1*-DMR. LD analysis was performed using SNPs (coded with 0, 1, and 2) called from the whole-genome sequencing (WGS) data and the mCG level of the *tb1*-DMR. The horizontal black line indicates the physical positions of the SNPs. (**b**) The SNP genotypes of teosinte and landrace samples around the Hopscotch transposon insertion region. Source data are provides as a Source Data file.

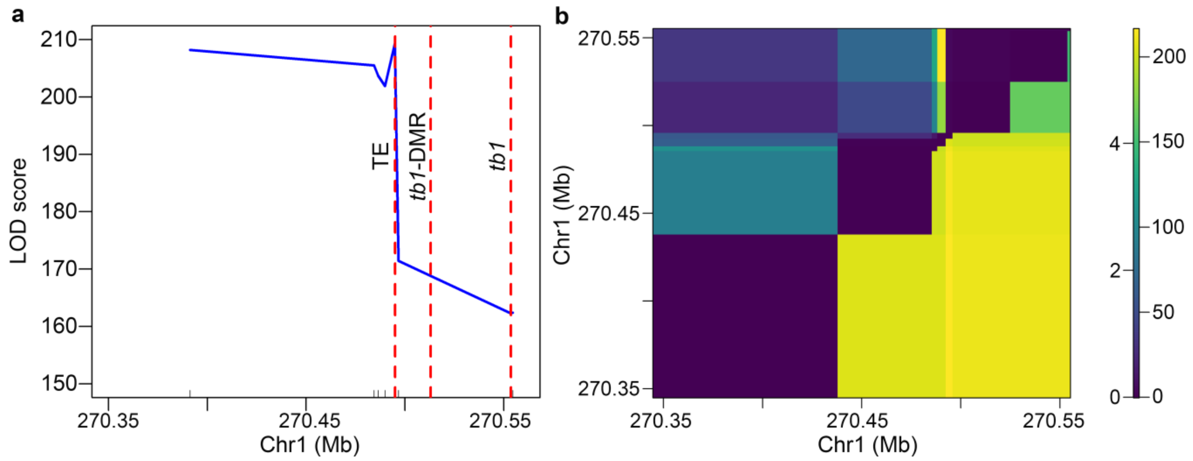

Supplementary Fig. 23. The quantitative trait locus results for the tillering phenotype around *tb1* locus. (**a**) The conventional single-QTL mapping result. Ticks above the x-axis indicate the physical positions of the SNP markers. (**b**) The two-dimensional QTL scanning result. The lower diagonal denotes the logarithm of the odd (LOD) scores for the joint two-locus QTL scanning; the upper diagonal denotes the LOD scores for the epistasis of the two loci. The color scale on the right indicates LOD scores for the joint two-locus (right) and epistasis (left) results.

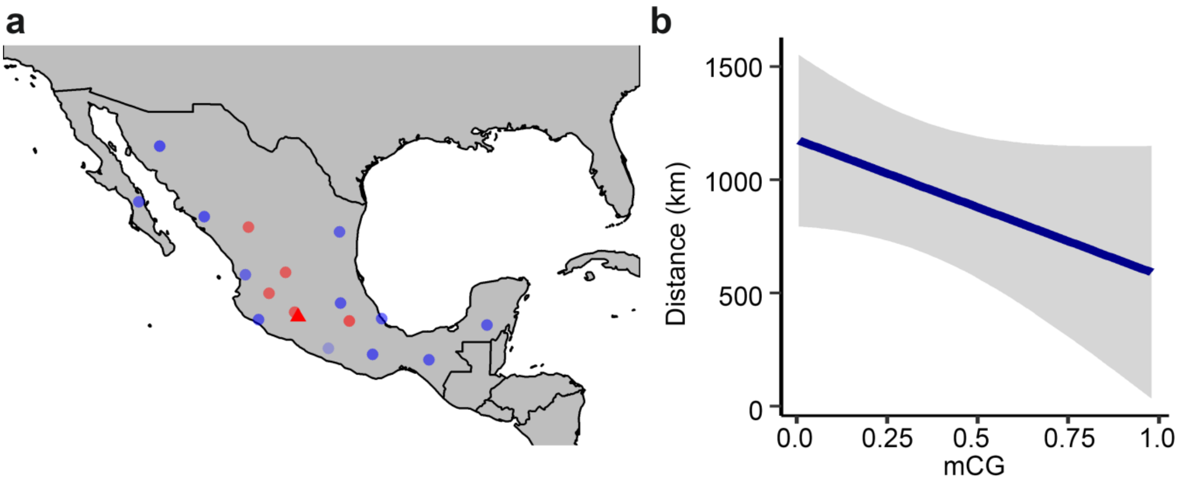

Supplementary Fig. 24. Correlation analysis between geographical distributions of landrace samples and their CG methylation levels at *tb1*-DMR. (**a**) Geographical distributions of the teosinte (red triangle) and landrace (points) samples. Red color denotes highly methylated and blue color denotes lowly methylated samples in the *tb1*-DMR. (**b**) The levels of mCG correlated with the distances to the origin (Balsas River Valley) of maize. Dark blue line indicates the regression lines and the shading area marks the 95% confidence band.
