# Supplementary material for: Evolutionary and functional genomics of DNA methylation in maize domestication and improvement
Source: Nat Commun. 2020 Nov 2;11:5539. doi: 10.1038/s41467-020-19333-4 (PMC7606521; doi:10.1038/s41467-020-19333-4)
Supplement: Supplementary file 3 — Description of Additional Supplementary Files [file 41467_2020_19333_MOESM3_ESM.pdf]

Description of Additional Supplementary Files

File Name: Supplementary Data 1
Description: Teosinte, landrace, and modern maize samples used in the study.

File Name: Supplementary Data 2
Description: Population-wide DMRs in the CG and CHG contexts.

File Name: Supplementary Data 3
Description: Selective sweeps detected between populations.

File Name: Supplementary Data 4
Description: Linkage disequilibrium (LD) analysis between DMR and local SNPs.

File Name: Supplementary Data 5
Description: The list of genes with CG teosinte-maize DMRs located at the exonic regions.

File Name: Supplementary Data 6
Description: The list of genes exhibiting interactive loops between genes and hypomethylated DMRs in maize located at the intergenic regions.

File Name: Supplementary Data 7
Description: Flowering time candidate genes located at sweep DMRs and interacting DMRs.
